# Case Report: Morbihan disease treated with tofacitinib successfully

**DOI:** 10.3389/fimmu.2023.1177316

**Published:** 2023-06-05

**Authors:** Zi-Yun Li, Chao-Cheng Chi, Sui-Qing Cai

**Affiliations:** Department of Dermatology, The Second Affiliated Hospital of Zhejiang University School of Medicine, Zhejiang, China

**Keywords:** JAK inhibitor, tofacitinib, Morbihan disease, case report, inflammation

## Abstract

**Introduction:**

To date, there is no standard treatment for Morbihan disease. Several studies have reported that Morbihan disease responds well to systemic corticosteroids (prednisone and prednisolone), systemic antibiotics (tetracyclines), antihistamines (ketotifen) and surgical therapy (Lymphaticovenous anastomosis). To our knowledge, Tofacitinib, as a Janus-activated kinase (JAK) inhibitor, plays a vital role in the treatment of inflammatory and autoimmune disorders. Therefore, Tofacitinib may be a promising medical option for patients with Morbihan disease.

**Case Presentation:**

The first case involves a 43-year-old Chinese man who presented a 12-month history of progressive painless swelling of the left upper eyelid. According to the skin biopsy, perivascular dermal edema with dilatation of lymphatic vessels and telangiectasia was observed, accompanied by mixed lymphocyte infiltrate, including histiocytes, plasma cells, and a few eosinophils. The second case involves a Chinese female patient who presented a 2-year history of progressive left-sided facial edema, which was eventually diagnosed as Morbihan disease. The skin biopsy revealed lymphocyte infiltration in the superficial vessels of dermis and some accessories. Based on patients’ clinical presentation, skin biopsy results, and exclusion of differential diagnoses such as systemic lupus erythematosus (SLE), they were diagnosed with Morbihan disease. They were both treated with Tofacitinib (5mg, po twice daily).

**Outcomes:**

Patient 1 underwent a trial of Tofacitinib at a dosage of 5 mg twice daily for one month, with notable improvement. His edema and erythema present on the left face were alleviated. Patient 1 reduced the dosage of Tofacitinib by half (5mg, once daily) and continued using it for 5 months. During the 6-month follow-up, the facial erythema in the patient subsided, and there was a noticeable improvement in the swelling of the left eyelid compared to before. Patient 2, her lesions gradually improved after one-week treatment. She received a one-month treatment of Tofacitinib, and during the subsequent six-month follow-up, there was no evidence of eruption recurrence.

**Conclusion:**

We present the first cases of two patients receiving short-term Tofacitinib as therapy for Morbihan disease and retrieving huge succession. Tofacitinib may be a promising oral alternative for patients with Morbihan disease. However, its safety and efficacy require further assessment through clinical trials.

## Introduction

Morbihan disease is a rarely reported and poorly understood chronic idiopathic dermatosis. Clinical manifestations are permanent facial edema, and an erythematous, which typically affects the central region of the face. However, Morbihan disease lacks specific pathological results to diagnose or develop a well-tested treatment program. Tofacitinib is a Janus-activated kinase (JAK) inhibitor mainly used to treat inflammatory diseases such as rheumatoid arthritis, psoriatic arthritis, etc. In this article, we describe 2 cases of severe Morbihan disease treated in our dermatology department with Tofacitinib, 5mg, twice daily.

## Case reports

### Case 1

**Figure 1:**
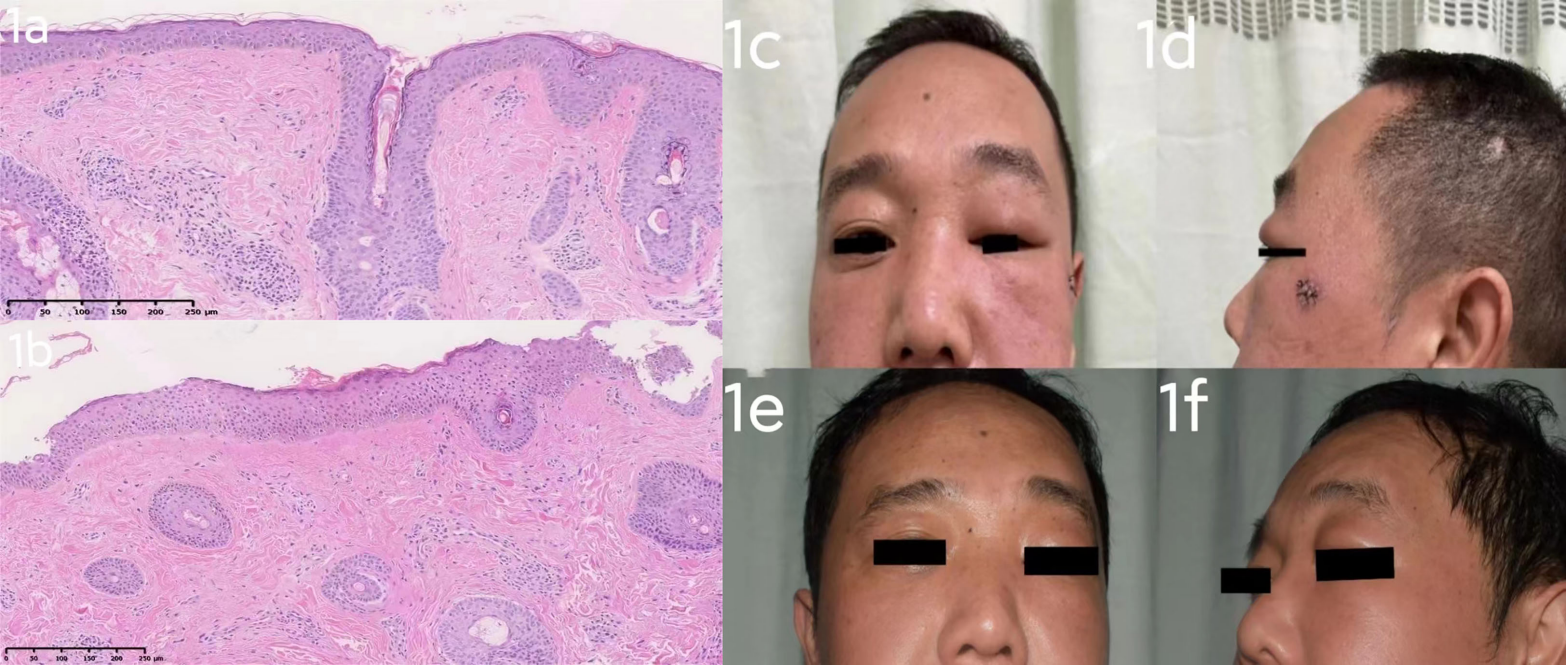
**(A)**: Several follicular ostia, mild sebaceous hyperplasia, superficial dermal telangiectatic vessels, as well as perivascular and perifollicular inflammation. Perivascular granulomas were also detected. (Hematoxylin-eosin stain; ×10). **(B)**: Pathological examination shows an unusual proliferation of abundant elastic fibers and perivascular infiltration by inflammatory cells. (Hematoxylin-eosin stain; ×10). **(A, B)** The excisional biopsy of the patient 1 support a diagnosis of Morbihan disease. **(C, D)**: case 1 before Tofacitinib treatment: diffuse erythema and redness on his cheeks, and his left eyelid displayed evident erythema, swelling, and ptosis. **(E, F)**: case 1 after 6 months’ Tofacitinib treatment: erythema and redness on his cheeks has subsided, there was a noticeable improvement in the swelling of the left eyelid compared to before.

**Figure 2:**
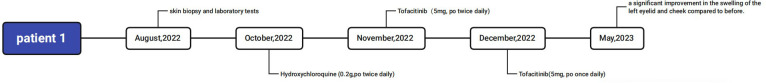
The timeline of laboratory investigations and ongoing treatments. (case1).

Patient 1 (**Figure 1**) was a 43-year-old male who presented to the clinic with a 12-month history of progressive pain and swelling in his left face and left upper eyelid. Examination revealed diffuse erythema and redness on his cheeks, and his left eyelid displayed evident erythema, swelling, and ptosis. The patient did not report any connection between his condition and alcohol consumption, spicy food intake, emotional stress, or medication. He had no constitutional symptoms and was in good health previously. In particular, he has no identified risk factors (non-smoker/non-drinker) and has no history of prolonged drug use or drug allergies. There is no history of blurred vision, muscle weakness, fever, and chest tightness. He had been previously diagnosed with rosacea of the granulomatous type and had been intermittently treated with oral doxycycline, which showed only slight effectiveness in reducing the patient’s facial redness. A differential diagnosis between dermatomyositis and systemic lupus erythematosus (SLE) was considered and a skin biopsy was performed in conjunction with blood testing. The presence of anti-double-stranded DNA (dsDNA) antibodies, anti-neutrophil cytoplasmic antibodies (ANCA), anti-extracted nuclear antigens (ENA) autoantibodies and antinuclear antibodies (ANA), creatinine kinase, aldolase, and full blood count results were within normal range. Recently excised biopsies of the patient revealed perivascular dermal edema with dilatation of lymphatic vessels and telangiectasia, accompanied by a mixed lymphocyte infiltrate that included histiocytes, plasma cells, and a few eosinophils. Furthermore, perivascular granulomas were detected in the biopsy (**Figure 1A, B**), leading to the diagnosis of Morbihan disease. Initially, the patient was prescribed Hydroxychloroquine (0.2g, po twice daily) for one month, but it was found to be ineffective. Therefore, the patient underwent a trial of Tofacitinib at a dose of 5 mg twice daily for one month, which resulted in a notable improvement. The edema and erythema present on the left face were alleviated. Subsequently, we halved the dosage of Tofacitinib (5mg, po once daily) (**Figure 2**). We conducted a follow-up on patient 1 and after receiving a six-month treatment of Tofacitinib, the swelling and redness on the left cheek of the patient has disappeared (**Figure 1E, F**), and there has been a significant improvement in the swelling of the left eyelid compared to before.

### Case 2

**Figure 3:**
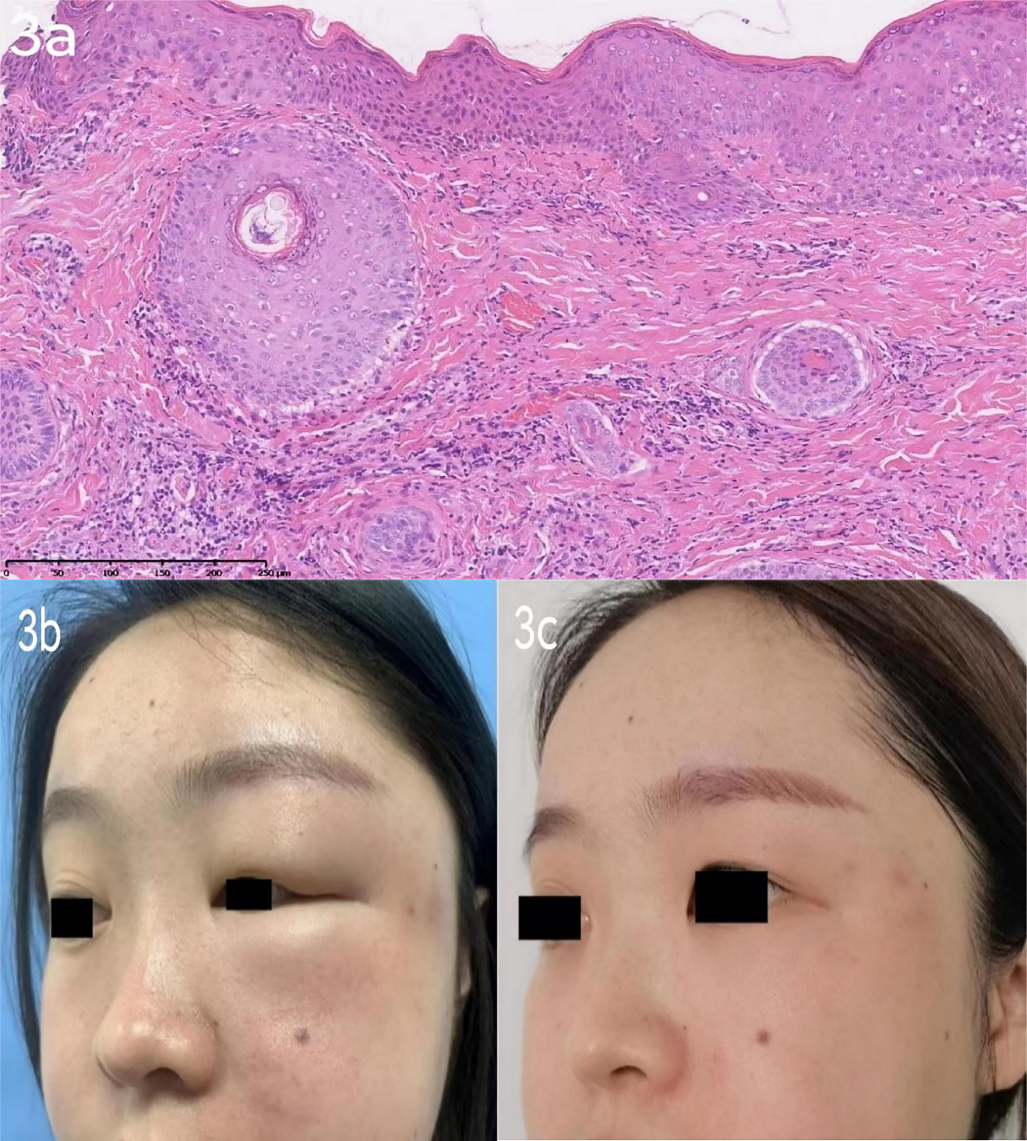
**(A)**: Patient 2 excision biopsy [left face]: lymphocyte infiltration in the superficial vessels of dermis and some accessories. (HE×10). **(B)**: case 2 before Tofacitinib treatment: Solid persistent eyelid and facial edema could be found on the left side face. **(C)**: case 2 after Tofacitinib treatment:The swelling of the left eyelid and redness of the left cheek have subsided.

**Figure 4:**
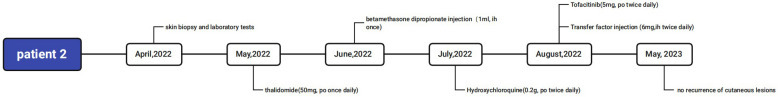
The timeline of laboratory investigations and ongoing treatments. (case2).

Patient 2 (**Figure 3B**) was a 29-year-old Chinese female patient who came to the clinic with a 2-year history of progressive left-sided facial edema. Physical examination revealed diffuse erythema and edema of the left eyelids, nonpitting edema, and flushing of the left cheek and nose, and erythematous papules over the left cheek region. The pronounced eyelid edema resulted in partial ptosis of the left eyelid. The patient had no significant medical history, including rosacea or allergies. The patient’s haematologic results, such as ANCA, dsDNA, ANA, creatinine kinase, were also unremarkable. The patient did not report symptoms of muscle weakness, joint pain, or blurred vision. The patient was recently admitted to the dermatological department, and an excision biopsy revealed lymphocyte infiltration in the superficial vessels of the dermis (**Figure 3A**). Based on her skin biopsy and clinical findings, the dermatologist diagnosed her with Morbihan disease. In the following months, the patient firstly accepted Thalidomide (50mg, po once daily) as her first-choice treatment and the result was dissatisfied. The patient was then placed on betamethasone dipropionate injection (1 ml, ih once). Considering the patient’s doubt regarding the use of hormones by her doctor, the treatment was modified to tofacitinib (5 mg, po twice daily) and transfer factor (TF) injections (6 mg, ih twice weekly) following discharge. After one-week treatment, facial swelling was assessed as moderate by her doctor (**Figure 3C**). Subsequently, the patient continued to use tofacitinib for one month, and during the subsequent six-month follow-up, there was no recurrence of cutaneous lesions (**Figure 4**).

## Discussion

Morbihan disease is a syndrome of unknown etiology that affects the upper half of the face. The term Morbihan, derived from the name of a district in Brittany, France, was first used by Robert Degos in 1957 to describe the first French patient observed with this condition (1). Historically, Morbihan disease has been considered a clinical variant or complication of acne or rosacea. Yet, the patient of case 2 has shown irrelevant clinical presentation to rosacea. The histopathological examination of this case remains vague, characterized by dermal swelling, perivascular and periadnexal lympho−histiocytic infiltration, with more or less numerous mast cells, and dilated lymphatic vessels (2).

The differential diagnosis of chronic facial edema is extensive and encompasses a variety of disease (3)(e.g. SLE, angioedema, allergic dermatitis, and dermatomyositis), infectious (e.g. erysipelas and herpes zoster), congenital (e.g. facial hemiatrophy and Apert’s syndrome), and malignant (e.g. angiosarcoma, lymphoma, and mycosis fungoides). The key distinguishing feature of Morbihan disease from these disorders is the absence of systemic manifestations and laboratory abnormalities. Therefore, a thorough clinical history and complete physical examination are crucial for the early diagnosis of this disease (4).

The up-to-date management of Morbihan disease is primarily divided into two categories: pharmacological treatment and surgical intervention. Recommended medical options for Morbihan disease include systemic corticosteroids (prednisone and prednisolone), systemic antibiotics (tetracyclines), antihistamines (ketotifen), thalidomide and isotretinoin (4). Ketotifen (3) is a potent H1-antagonist that exhibits antiallergic activities by inhibiting the release of mediators from mast cells. This mechanism of action is believed to be responsible for its efficacy in preventing fibrosis, which is one of the possible causes of Morbihan disease. Due to the anti-angiogenic, anti-inflammatory, and immunosuppressive effects of thalidomide, some literature mentions its application in inhibiting the inflammatory response of Morbihan disease. However, in the case of our female patient, thalidomide showed poor efficacy and was subsequently discontinued. Chaidemenos et al. (5) recommended that long-term Minocycline monotherapy may be appropriate when histology sections reveal significant mastocyte infiltration. They observed that doxycycline administered in doses of 200 mg/day for 4–12 months in five patients was effective, but short-duration treatment was irreflective whether mastocyte infiltrated or not. Shim et al. (6) reported successful treatment with doxycycline and colchicine, with a treatment cycle of 7 months and recurrence during a 14-month follow-up. Katharina et al., discovered that the combination of ultra-low-dose isotretinoin with antihistamines yields remarkable therapeutic efficacy and minimal adverse effects in the treatment of Morbihan disease. Despite the limited knowledge regarding the pathogenetic relationship between Morbihan disease and Rosacea, there appears to be an association. Isotretinoin has been recognized for its significant efficacy in alleviating symptoms of Rosacea due to its immunomodulatory and anti-inflammatory properties (7). Antihistamines, such as desloratadin, not only reduce histamine production but also inhibit the secretion of IL-6 and IL-8 from mast cells. Furthermore, one of antihistamine side effects is a reduction in adipogenesis, while isotretinoin, on the other hand, has a significant side effect of causing hyperlipidemia. Therefore, when these two medications are combined, they can generate minimal adverse effects. In cases where non-surgical therapy fails to control symptoms of lymphoedema, surgical interventions such as Lymphaticovenous anastomosis (LVA) and eyelid reduction surgery should be considered. In 2021, Hattori (8) first showed good results in applying Lymphaticovenous anastomosis in Morbihan disease. Novel treatment options include omalizumab, as Kafi et al. (9) successfully explored its use as a therapeutic option. They hypothesized that omalizumab could successfully stabilize mast cells in Morbihan disease and reduce temporary swelling by decreasing Fc receptors on the cell surface and binding to the circulating IgE (10).

The underlying cause of Morbihan disease remains undetermined. One potential theory is based on the chronic inflammatory process accompanying this disease. This process may lead to the destruction of elastin surrounding blood vessels, resulting in transudation of fluids, or it may cause fibrosis and permanent obstruction of deep dermal lymphatic vessels with fluid accumulation, congestion and lymphoedema of the underlying tissue (11). As a Jak inhibitor, Tofacitinib can directly influence molecules that drive cutaneous changes (IL-19, IL-22, IL-24). Therefore, Tofacitinib may help to suppress the immune response causing inflammation and damage in Morbihan disease (12). While our case did not present any adverse reactions and infections, further investigation is necessary to determine long-term safety and efficacy of Tofacitinib.

Our study has several limitations. Firstly, the findings are based on two cases, and therefore may not be generalizable. Secondly, as this is a report on the treatment of Morbihan disease with Tofacitinib, we cannot ascertain the molecular mechanism of the treatment. Further relevant studies are necessary to draw definitive conclusions

## Conclusion

To the best of our knowledge, we present the first cases of two patients receiving Tofacitinib as therapy for Morbihan disease and retrieving huge succession. Tofacitinib may be a promising medical option for patients with Morbihan disease. However, the long-term safety and efficacy of Tofacitinib require further assessment through clinical trials.

## Data availability statement

The original contributions presented in the study are included in the article/supplementary material. Further inquiries can be directed to the corresponding author.

## Ethics statement

The studies involving human participants were reviewed and approved of the Second Affiliated Hospital Of Zhejiang University School Of Medicine. The two patients in this manuscript have given written informed consent for the publication of their case details.

## Author contributions

ZL and CC collected the data and drafted the manuscript. S-QC conceived of the study, and participated in designing, writing, reviewing, and revising this manuscript. All authors contributed to the article and approved the submitted version.
